# A Goal Without a Plan Is Just a Wish—Creating a Personalized Aftercare Plan for Breast Cancer Patients Supported by the Breast Cancer Aftercare Decision Aid

**DOI:** 10.3390/curroncol32100552

**Published:** 2025-10-01

**Authors:** A. Dekker-Klaassen, C. H. C. Drossaert, R. Thé, A. M. Zeillemaker, M. van Hezewijk, I. M. De Keulenaar-Suiker, B. J. Knottnerus, A. Honkoop, M. L. van der Lee, J. C. Korevaar, S. Siesling, on behalf of the NABOR Project Group

**Affiliations:** 1 Department of Health Technology and Services Research, Technical Medical Centre, University of Twente, 7500 AE Enschede, The Netherlands; 2 Department of Research and Development, Netherlands Comprehensive Cancer Organisation (IKNL), 3511 LC Utrecht, The Netherlands; 3 Department of Psychology, Health & Technology, University of Twente, 7500 AE Enschede, The Netherlands; 4 Department of Development, ZorgKeuzeLab, 2611 BN Delft, The Netherlands; 5 Department of Surgery, Alrijne Hospital, 2334 CK Leiden, The Netherlands; 6 Radiotherapiegroep, Institution for Radiation Oncology, 6800 JD Arnhem, The Netherlands; 7 Department of Surgery, Noordwest Ziekenhuisgroep, 1815 JD Alkmaar, The Netherlands; 8 Netherlands Institute for Health Services Research (Nivel), 3500 BN Utrecht, The Netherlands; 9 Department of Internal Medicine, Isala Clinics, 8025 AB Zwolle, The Netherlands; 10 Scientific Research Department, Helen Dowling Institute, Centre for Psycho-Oncology, 3723 MB Bilthoven, The Netherlands; 11 Department of Medical and Clinical Psychology, Tilburg University, 5000 LE Tilburg, The Netherlands; 12 Faculty of Health, Nutrition and Sport, The Hague University of Applied Science, 2521 EN The Hague, The Netherlands

**Keywords:** breast cancer, aftercare, shared decision making, aftercare plans, survivorship, personalization

## Abstract

**Simple Summary:**

Breast cancer patients often face long-term challenges after completing treatment, such as tiredness, pain and fear of cancer recurrence. Personalized aftercare can help them manage these issues, but such care is not always tailored to individual needs. This study describes the development of the Breast Cancer Aftercare Decision Aid (BC-ADA), a tool designed to support shared decision-making between patients and healthcare professionals. The tool provides information on five key areas of recovery—physical health, emotions, relationships, trust in the body, and resuming daily activities. Patients can indicate their goals and support needs, which helps create a personalized aftercare plan together with their healthcare professional. The tool was developed with input from both patients and healthcare professionals and was found to be user-friendly and relevant. The BC-ADA has the potential to improve aftercare by promoting personalized, goal-based recovery support and is currently being tested in clinical practice.

**Abstract:**

Aftercare plans can support breast cancer patients’ self-management after curative treatment but are often not personalized and limitedly applied by healthcare practitioners (HCPs). This study aimed to develop a tool integrating information provision and assessment of patients’ goals and needs, to support the creation and application of a personalized aftercare plan. A multidisciplinary workgroup guided the development by defining the target audience, scope and purpose. Needs of 18 patients and 15 HCPs were assessed to determine the tool’s content and format. Usability tests of a prototype among 7 patients and 10 HCPs informed improvements and finalization. The tool, called ‘Breast Cancer Aftercare Decision Aid’ (BC-ADA), provides information on potential effects of cancer and support options on five domains: physical wellbeing, emotions, relationships, regaining trust and return to daily routine. Patients can indicate which domain(s) they wish to improve, what resources they have and where additional help is needed. Based on their answers, patients can create an aftercare plan together with the HCP, including personal goals, specific actions and agreements on follow-up. Usability and acceptability were positively evaluated by both patients and HCPs. The BC-ADA seems promising in supporting personalized aftercare decision-making and is currently being tested in the NABOR-study in Dutch hospitals.

## 1. Introduction

Breast cancer survivors often face challenges as they navigate through life after curative treatment. Both physical and psychosocial complaints, such as tiredness, pain and fear of cancer recurrence, can persevere for more than five or ten years after treatment and hinder patients’ daily functioning [1,2]. The variety and severity of complaints differ among patients, leading to diverse care needs [1,3,4,5,6,7]. This underscores the importance of personalized aftercare to optimally align with patients’ needs and support self-management during recovery [3]. However, facing a myriad of available support options and self-help resources can make it challenging for patients and their healthcare practitioners (HCPs) to determine the appropriate type and intensity of aftercare needed.

Aftercare plans—online or printed documents, usually including a treatment summary, information on management of complaints, and agreements on the aftercare trajectory—are crucial for aligning care with patient needs and promoting self-management in cancer survivorship [8,9,10,11,12,13,14]. However, current implementation is limited [11,15] and existing aftercare plans are often not personalized to the patients’ individual needs [16,17]. Previous studies also reveal that many patients feel inadequately supported and informed about the potential impact of their cancer and treatment and available support [18,19]. Recent findings show that in Dutch hospitals, patients’ needs are often not actively assessed, leading to random aftercare variations that depend on patients’ assertiveness to ask for help instead of being based on well-informed, shared decisions [16]. This lack of personalization contrasts with patients’ desires for aftercare. According to patients in our previous study [17], their most important care needs should be better identified and structurally addressed during the aftercare consultation with their HCP. They desired that—based on these discussions of care needs—they could make shared decisions with their HCP about the support needed and self-management steps. These decisions can be summarized in an aftercare plan, which patients perceived as a valuable tool to provide greater clarity regarding their aftercare trajectory and facilitate evaluation of their recovery.

Aftercare plans should be created in shared decision-making (SDM) to ensure that patients and HCPs make informed decisions that reflect patient’s personal needs and preferences [20,21]. Patient Decision Aids (PtDAs) are effective tools in supporting SDM, as they provide patients with structured, accessible information and encourage collaborative discussions with their HCPs [22]. Several PtDAs have already been developed within survivorship care [23], including two PtDAs for breast cancer survivors [24,25]. One PtDA supports SDM on the intensity of surveillance (with regular mammograms or other imaging techniques) to detect breast cancer recurrences but does not focus on aftercare [25]. The other PtDA focused on follow-up care in general, encompassing both surveillance and aftercare, and aims to assist breast cancer survivors in making choices about the frequency of follow-up consultations [24]. To the best of our knowledge, no tool has yet been developed that focuses specifically on personalization of aftercare and addresses patients’ information and care needs.

Therefore, our study aims to develop a PtDA that supports shared decision-making for a personalized aftercare plan. By integrating relevant and easily accessible information on the potential impact of breast cancer and treatment and support options, and assessment of patients’ personal goals, preferences, and care needs, the PtDA can empower patients and HCPs to collaboratively create personalized aftercare plans that optimize patients’ self-management throughout the survivorship journey.

## 2. Materials and Methods

The development of the PtDA was initiated by the University of Twente, The Netherlands Comprehensive Cancer Organization (IKNL) and The Hague University of Applied Science, in collaboration with Zorgkeuzelab, a company which develops PtDA’s for clinical practice. The process of the development of the PtDA was performed in co-creation with a multidisciplinary steering group, which we formed from HCPs involved in aftercare for breast cancer patients, patient representatives of the breast cancer organization in The Netherlands (BVN), researchers and web developers (Table 1).

**Figure 1:**
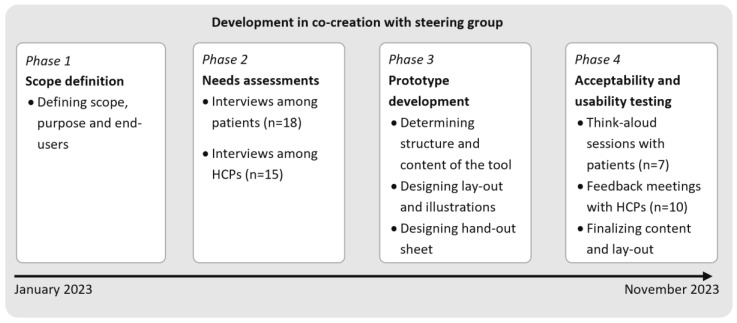
Phases of development of the PtDA.

The steering group convened in six co-creation sessions to reach consensus on the scope and content of the PtDA. The development process consisted of four consecutive phases (Figure 1), aligned with the International Patient Decision Aids Standards (IPDAS) [26] and informed by prior similar research [25].

Phase 1: Scope definition

In the first co-creation session, the steering group defined the scope, target users, and aims of the PtDA. This was based on consensus discussions among the group members. These agreements guided the needs assessment among patients and HCPs to evaluate the current aftercare system, identify opportunities for improvement, and determine the PtDA’s optimal timing and its role in decision-making about aftercare.

Phase 2: Needs assessments among patients and HCPs

Two required assessment studies were carried out: one among patients and one with HCPs. For both, the Medical Research Ethics Committees United confirmed that those were not subject to the Medical Research Involving Human Subjects Act (WMO), and ethical consent was applied (reference number W23.085).

We conducted semi-structured interviews among 18 patients from five different Dutch hospitals, who received curative treatment for primary invasive breast cancer and did not have metastases and did not carry gene mutations. Most of them received aftercare for one to three years since completion of curative treatment (i.e., surgery), and two participants completed their curative treatment six and 16 years ago. We included both women and men to ensure that also men’s needs were considered during development of the tool, since they also belong to the target users. The questions related to patients’ preferences concerning identification of care needs, information provision and the use of aftercare plans. A detailed description of the study population, data collection, and data analysis can be found in a previous publication [17].

To assess HCPs’ needs, interviews were conducted with 15 HCPs from ten Dutch hospitals: seven nurse specialists, seven nurse practitioners and one surgical oncologist. We sent invitations to each hospital team, asking for two HCPs highly involved in aftercare, including at least one nurse specialist or nurse practitioner, to participate in a group-interview. Group-interviews took place in three hospitals, in seven hospitals HCPs were interviewed individually, since HCPs were unable to find a mutually convenient time. HCPs were asked about their experiences and desired improvements regarding (1) ways to assess patients’ individual care needs, (2) information provision about breast cancer impact and options for self-help and support, (3) personalized aftercare plans and (4) potential barriers and facilitators for implementation of a PtDA. HCPs also provided opinions on existing tools to assess care needs, such as the Distress thermometer or Patient Reported Outcome Measurements (PROMs) [27,28], as well as ideas and wishes for a new tool to support personalization of aftercare. The full interview guide is included in Appendix A. Interviews took place in May and June 2023 and lasted for about 30 min. Interviews were audio recorded, with participants’ permission and transcribed verbatim. At the end of the interview, HCPs were asked for permission to recontact them in the future to give feedback on a prototype of the tool. Two independent coders (ADK and LF) performed thematic analyses using Atlas.ti 24, by identifying perspectives on and preferences for personalization of aftercare in accordance with the main interview topics. The analyses were discussed with a third researcher (CHCD) until consensus was reached.

Phase 3: Prototype development

Findings from the needs assessments were discussed with the steering group and used to develop a list of requirements. The following questions were central to the discussions with the steering group: How can the PtDA facilitate shared decision-making about the patients’ recovery and care needs? What information (i.e., about the potential impact of breast cancer and treatment and support options) do the patients need to enable shared decision-making regarding the continuation of the aftercare trajectory? These questions were deliberated by the steering group members using three fictional patient cases (personas) as examples. Based on these discussions and the results from the needs assessments, a first prototype of the online tool and hand-out sheet of the PtDA was developed. Then, the prototype was presented in iterative cycles to the steering group, who reviewed and revised the medical content and structure and provided feedback on the lay-out and illustrations of the prototypes, until consensus was reached.

Phase 4: Usability and acceptability testing

The usability and acceptability testing was conducted via online meetings of 30 min. All patients and HCPs who participated in the previous needs assessments were invited to take part in the usability and acceptability study. Participating HCPs were given background information about the PtDA’s development and asked to review it independently. The researcher (ADK) then discussed each component with them, noting feedback on content, layout, usefulness, and timing. The patients received login instructions and an example of the handout sheet via email, before they participated in online meetings to provide feedback.

Usability was assessed through ‘Think Aloud’ sessions, where patients were asked to use the PtDA while verbalizing their experiences, thoughts and suggestions [29]. The researcher (ADK) observed their interaction with the tool and identified issues through probing questions, such as “*Why do you find this interesting?*” or “*You look confused; what are you thinking?*”. HCPs were asked to provide feedback on the usability of the information and questions for their patients and the usefulness of the summary in aftercare consultations.

Acceptability was evaluated regarding the extent to which users perceive the PtDA as understandable and well-balanced in its length, amount of information and presenting options. During the online meetings with patients and HCPs, feedback was collected on ease of use, length and amount of information and perceived relevance of questions. Patients were also asked if and why they were (dis)satisfied and if they would recommend this PtDA to other patients. All feedback of patients and HCPs on each component of the PtDA was listed and subsequently addressed if either patients or HCPs or both were not satisfied with the content.

## 3. Results

Phase 1: Scope definition

The PtDA focuses on aftercare, for both men and women who have completed curative treatment for breast cancer and are transitioning back to their daily routine. In order to provide personalized support from HCPs in hospital settings (mainly nurse practitioners and nurse specialists), the PtDA should provide insight into their patients’ individual care needs, increase patients’ knowledge on self-help and support options, and facilitate patients and HCPs in the creation of a personalized aftercare plan.

Phase 2: Needs assessments among patients and HCPs
Phase 2.1. Needs and wishes of patients

In total, 18 patients (15 female, 3 male) participated, with a mean age of 56. Most of them received radiotherapy and/or hormonal therapy and almost half of the patients received chemotherapy. The results of the needs assessment study among patients have been published in detail elsewhere [17]. In summary, patients emphasized the importance of HCPs addressing not just physical complaints but also needs on other domains, including emotional and mental wellbeing, relationships and daily functioning. They also expressed a need for a ‘positive approach’, focusing on the improvements they want to achieve rather than their complaints and limitations in daily practice. From their HCPs, patients valued empathy, time, and probing to decrease any inhibitions in sharing their concerns. Most patients also expressed a wish to prepare themselves for aftercare consultations by reflecting on their care needs beforehand. Patients differed in their preferences regarding the intensity of aftercare and desired that these preferences would be actively assessed and discussed with their HCP. Most patients expected that an aftercare plan, summarizing their received treatment(s), agreements about the future aftercare trajectory and the next follow-up steps in their recovery, would be valuable. Some patients also considered an aftercare plan as a useful tool for external communication with HCPs outside the hospital, such as occupational health doctors or general practitioners (GPs). A few patients expressed that—even though they wanted to discuss their wishes for improvement with their HCP—they did not need an aftercare plan, since they could manage their recovery independently.

Patients also highlighted the importance of easily accessible information that could be consulted when needed, to prevent feeling overwhelmed by information. They desired specific and comprehensive information about: the expected recovery, potential late effects of cancer and treatment, the available support options, and how to self-manage specific complaints.

Patients were generally positive about the concept of a PtDA that assesses care needs and provides information and expressed different potential benefits of such a PtDA (Table 2).

Phase 2.2. Needs and wishes of HCPs

HCPs expressed the need for better prioritizing patients’ care needs across different domains, to focus the aftercare consultation effectively. A positive approach was preferred, as one HCP stated: “*You don’t want to list complaints but rather ask: what do you need to regain the life you had before?*”. They also wished that a tool would help patients explain what they want to improve and the steps they could take to achieve this. Additionally, they valued having a clear overview of care needs themselves, to help them prepare for the consultation. Some HCPs questioned whether more needs would be identified when assessing care needs beforehand: “*There’s a chance patients will focus more on their symptoms, which could lead them to seek additional care. Some may then become overly focused on it, while others may feel particularly well-heard*”.

HCPs emphasized the importance of informing patients about the aftercare trajectory and the potential impact of breast cancer and its treatment across various domains but mentioned that it is challenging to deliver comprehensive information within the time constraints of an aftercare consultation. Therefore, they preferred to refer their patients to a PtDA that includes a clear overview of practical tips for managing symptoms, options for further support, and referrals to other reliable resources and websites.

HCPs expressed willingness to use an aftercare plan, provided it was concise and summarized only the decisions made during the aftercare consultation regarding following aftercare appointments and the patient’s planned actions to achieve their goals: “*Summarizing briefly: what are the following steps we assign as ‘homework’? This allows the patient to review them later and, hopefully, follow through*”. While some HCPs believed the aftercare plan should also include information on the patient’s tumor and treatment characteristics and contact details of HCPs, others felt this information was unnecessary as patients already have access to this information or can find it in their digital patient records. Two HCPs questioned whether patients would refer to the aftercare plan after the conversation: “*I have no doubt about its usefulness. I just don’t know if they’ll read it again afterward*”.

All HCPs were generally positive about the concept of a PtDA that assesses care needs and provides information on managing the potential impacts of cancer and its treatment. They expressed several potential benefits of such a PtDA (see Table 2).

Phase 2.3. Potential barriers and facilitators for implementation of the PtDA

The most frequently mentioned barrier by HCPs was that such a online tool would not be suited for some patients, for instance, those with lower health literacy, limited digital skills, or those who do not speak Dutch. Additionally, two HCPs noted that their limited consultation time might prevent them from adequately discussing the PtDA (N = 2), and that their patients might perceive completing the PtDA as an additional burden, given that they are already fill out PROMs (N = 2).

HCPs also indicated several factors that would facilitate implementation. The majority mentioned that they were personally motivated to use the PtDA (N = 8), felt that it aligned well with their current routine of information provision and needs assessments (N = 5) and the hospital’s vision on patient-centered care (N = 2), and anticipated sufficient support among colleagues to adopt the PtDA in their workflow (N = 2).

Phase 3: Prototype development

**Figure 2:**
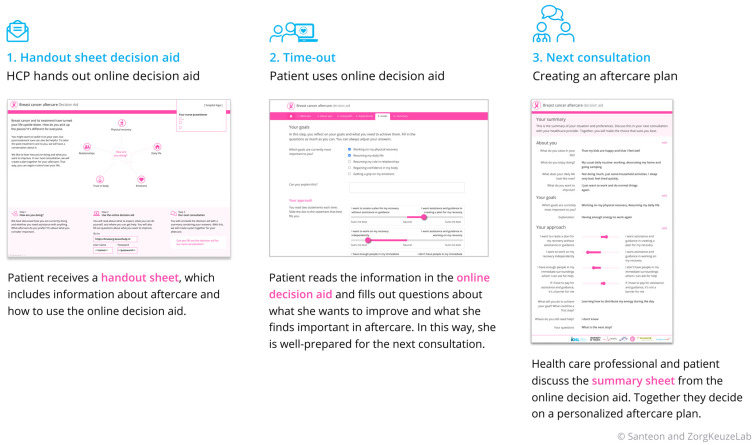
Three components of the tool.

The steering group decided to develop a PtDA which we called the ‘Breast Cancer Aftercare Decision Aid’ (BC-ADA) and which comprises three components, (1) a printed handout sheet to be handed to the patient by the HCP (2) an online tool, that the patient can use at home and (3) a summary sheet, that can be printed and/or sent to the HCP and serves as a conversation starter for the next conversation (Figure 2). Based on the expectations and needs of patients and healthcare providers, several requirements were established that the decision aid should meet, which were incorporated in the decision-aid (see Supplementary Table S1).

Component 1: handout sheet

The handout sheet is used by the HCP to introduce the tool to the patient and explain how aftercare can support recovery across various domains of life (see Supplementary Figure S1). The handout sheet also includes practical instructions for the patient on how to log in to the tool.

Component 2: Online tool

**Figure 3:**
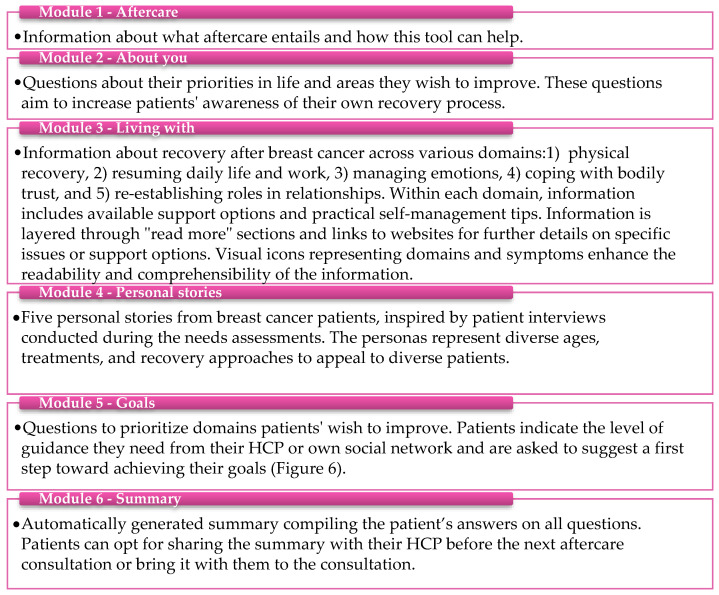
Content of the online tool in six modules.

The online tool of the BC-ADA comprises information about the potential impact of breast cancer and its treatment, options for self-help and support, and questions regarding the patient’s personal goals and care needs. This content of the tool is organized into six sequential modules (Figure 3), guiding patients to progressively understand their care needs, learn about self-help and support options, and ultimately set personal goals. In Module 3, the information is categorized into five different domains, which are based on frequent mentions in the needs assessments and the steering group’s identification of them as prevalent themes (Figure 3). Patients can access the information in the tool at their preferred timing and can adjust their answers on questions when preparing for future aftercare consultations. Screenshots of several modules are shown in Supplementary Figures S2–S4.

Component 3: The summary sheet

The summary is automatically generated in Module 6 of the online tool (Figure 3) and summarizes the patient’s personal goals and care needs, supporting the discussion about aftercare between the patient and the HCP. This summary serves as a conversation starter during the next consultation with the HCP (see Supplementary Figure S5 for an example summary). Discussing the summary leads to the creation of a personalized aftercare plan. Based on the goals and care needs as defined in the summary, the patient and HCP jointly decide on the necessary aftercare within the hospital or from external healthcare providers, actions the patient can take independently, and any follow-up appointments required.

Phase 4: Acceptability and usability testing

The steering group decided that the BC-ADA fulfills the requirements identified through the needs assessments (Supplementary Table S1), except for two requirements: we did not incorporate contact details of the hospital team as a standard element in the aftercare plan because some HCPs reported that patients already receive these details in another way (e.g., on business cards, online portals). Also, a link between the tool and patients’ electronic records could not be incorporated, as a secure connection could not be established to transfer the patient’s responses to their records. Instead, a link to the healthcare provider’s mailbox was created, enabling the patient to digitally share the summary with the provider via a button in the tool.

The acceptability and usability of the prototype were tested with seven patients and 10 HCPs (i.e., seven nurse specialists and three nurse practitioners) who also participated in the previous needs assessments; five HCPs declined due to time constraints and 11 patients did not respond to our invitation. The participating patients and HCPs generally had a positive first impression of the BC-ADA.

Phase 4.1. Perceptions of patients

All patients indicated that they would recommend the BC-ADA to other patients. Some patients mentioned that the BC-ADA provided a sense of control: “*It gives back a sense of agency, a bit of grip. You feel like you can take the lead yourself again*”, that it provided practical support: “*It’s nice that it provides tools to just move on, focusing on progress rather than dwelling on complaints or the fact that you were ill’’* and fostered a sense of recognition: “*This is so great! I see all the themes I struggle with, and those questions are really about me.*” Another patient anticipated that the BC-ADA would have been helpful in taking more initiative in her recovery: “*If I had received this tool, I would’ve benefited from it. I would’ve been better informed and might have discovered sooner what I could do myself.*” One patient noted that the BC-ADA had a different purpose than initially expected: “*I thought it would be about making decisions—like what referrals to make, how often to return. But it turns out to be more of a preparation for the conversation, something to make you more aware. That’s good, as long as the healthcare provider knows during the conversation exactly where to direct you for help*”.

During the Think-Aloud sessions, it was observed that patients used the information in Module 3 of the online tool differently; some thoroughly read all sections, while others skimmed through or focused on specific topics that appealed to them. Patients appreciated the ability to choose what to explore at their own pace: “*Relationships are not an issue for me, so I just skim that part*”; “*Work and financial consequences—I’m avoiding that for now, but it’s great that it’s included. If it becomes relevant, I know where to find it*”; “*This [information about body image concerns] is something I’m putting aside for now. It’s difficult for me, but I’ll look into it when I’m ready*”.

Phase 4.2. Perceptions of HCPs

Most HCPs also expressed positive views about the BC-ADA. A nurse practitioner remarked: “*Very well designed, aligns well with what we discuss in practice, and the included information fits with our vision of patient care.*” Another nurse practitioner praised the completeness and conciseness of the content: “*It’s great that such comprehensive topics are covered while keeping it concise.*” However, a nurse practitioner doubted whether the BC-ADA would achieve personalization: “*The approach is fantastic, aiming for more personalized care. But I wonder if this tool will truly accomplish that.*”

Regarding the timing of introducing the BC-ADA during the aftercare trajectory, HCPs indicated that it should be offered between three months to a year after surgery and that its exact timing depends on the duration of adjuvant treatment. For patients undergoing little to no adjuvant treatment, the BC-ADA should be introduced within three to six months post-surgery. However, for patients still undergoing adjuvant chemotherapy during this period, this would be too early, as they typically express a need for information and aftercare only after six months. Adjuvant hormonal therapy does not need to be considered regarding the timing of the BC-ADA, as hormonal therapy typically continues for several years post-surgery, while the BC-ADA can meanwhile assist patients with their aftercare needs.

Specific comments of patients and HCPs regarding each component of the tool were listed and changes were made if either patients or HCPs or both were not satisfied with the content (Supplementary Table S2), resulting in the final version of the BC-ADA.

## 4. Discussion

The Breast Cancer Aftercare Decision Aid (BC-ADA) was developed to support patients and healthcare professionals (HCPs) in creating personalized aftercare plans that address patients’ personal goals and care needs. Collaboratively designed with a multidisciplinary team, it is based on needs assessment studies among patients and HCPs. The BC-ADA meets all predefined requirements and is perceived as usable and acceptable by patients. This allows further evaluation of usability in a real-world hospital setting as is currently ongoing in the NABOR-study within Dutch hospitals [30].

The BC-ADA offers unique features compared to existing tools for breast cancer survivors, as it (1) integrates information provision and assessment of care needs; (2) adopts a positive approach; and (3) serves as a means to create an aftercare plan. Regarding the first feature, the integration of both information provision and needs assessment is important, as it may help patients to recognize potential care needs and goals, apply practical tips and prevent future care needs. Second, the positive approach is expected to encourage goalsetting aimed at improving patients’ overall physical, mental and social well-being [31,32], rather than focusing solely on symptom measurement, as seen in tools like the Distress Thermometer and PROMs [27,28]. By promoting achievable steps, the BC-ADA is expected to foster patients’ self-efficacy to attain their goals [33]. Recognizing that not all patients have the skills or social support to create and execute actionable plans, the BC-ADA allows patients to specify the level of support they need from HCPs and their social network. We did not include choice options regarding the number of aftercare consultations, as was included in the aftercare tool by Klaassen and colleagues [24], because that would detract from the primary focus on discussing whether and what type of aftercare (i.e., follow-up consultations, self-help tips or referral to other HCPs) is needed, which both patients and HCPs identified as the most valuable. In addition, letting patients determine how many aftercare consultations they prefer, may encourage their dependency on hospital-based care, which would hinder their self-management and lead to unnecessary healthcare utilization. Third, the BC-ADA’s summary sheet serves as a conversation starter for patients and HCPs to develop personalized aftercare plans. This summary may lower the threshold for patients to discuss care needs and enable HCPs to explore these needs more effectively. By using the summary to identify key goals, planned actions, and potential follow-up consultations, the resulting aftercare plan is more likely to align directly with the identified needs, enhancing its relevance and usefulness for patients [12].

An increasing number of tools is being developed but often underutilized in daily practice, partly due to poor alignment of the tool’s timing with existing care pathways and HCP’ limited self-efficacy and perceived usefulness of the tool [23]. Therefore, we invest substantial effort in embedding the implementation of the BC-ADA within the NABOR study [30], a large-scale trial in 10 Dutch hospitals investigating the (cost-)effectiveness of personalized follow-up after breast cancer. Using a multiple interrupted time series design, hospitals will stepwise transition from standardized care (without decision aids) to personalized care (with decision aids). To ensure successful implementation, we documented current aftercare pathways, provided HCPs with practical instructions about the decision aid, advocated e-learning modules about shared decision-making, and designated a local ambassador within each hospital who monitors progress and addresses any issues that need to be solved. Beginning in 2024, all 10 hospitals currently implement the BC-ADA in daily practice. Evaluation is still ongoing within the NABOR-study: patient-reported outcomes (e.g., shared decision-making, cancer worry, quality of life, patient satisfaction) are collected before and after BC-ADA implementation and the uptake and appreciation of the BC-ADA will be evaluated among patients and HCPs [30].

One of the key strengths of the BC-ADA is its iterative development process. The multidisciplinary steering group continuously aligned the BC-ADA’s content with the findings from needs assessment studies, ensuring a consistent approach that meets user needs and integrates seamlessly into daily practice. The involvement of diverse patients—both in the needs assessments and co-creation sessions—enhances the BC-ADA’s relevance and broad applicability. Notably, unlike other breast cancer tools [24], this one includes the needs of male patients, making it more inclusive. Another strength lies in the extensive consultation with HCPs across 10 different Dutch hospitals to understand care pathways and preferences. This collaboration informed the creation of a robust implementation strategy, and as an additional advantage, these HCPs serve as local ambassadors, promoting the BC-ADA within their teams. Moreover, the BC-ADA’s development and implementation within the NABOR study, which simultaneously investigates its cost-effectiveness, increases the likelihood of its sustainable use in daily practice and future improvements.

The study also acknowledges several limitations, including a small number of participants in usability testing and potential bias from involving previously engaged patients as they might have been positively predisposed or inclined to provide socially desirable feedback in the presence of the researcher. Therefore, our future evaluation of the BC-ADA will involve a larger, more representative group of patients who received the BC-ADA but were not previously engaged in its development. Another limitation is that the BC-ADA is less suitable for non-Dutch speaking patients and requires digital skills or resources to use the online tool. Although the BC-ADA incorporates visual elements to enhance usability, potential improvements could include multilingual text or audio options. Future studies should explore the needs of patients from diverse linguistic, cultural, and socioeconomic backgrounds, who often face greater healthcare navigation challenges and may require different support approaches than the current BC-ADA provides [34]. This follow-up research could also be conducted in other countries to explore how a similar decision aid supports personalized aftercare.

### Practical Implications

As argued above, our practical implications are as follows: (1) the BC-ADA can be implemented and further tested in a real-world hospital setting, where its effect on patient satisfaction and shared decision-making should be investigated; (2) the reach of the BC-ADA can be improved by offering it in multiple languages and studying how it aligns with different cultures; (3) the suitability and added value of the BC-ADA can also be explored in other countries. It is important to mention that the results regarding the effectiveness and usability of the tool from the NABOR study should first be awaited, as these may reveal the need for further refinement and improvement. After these findings become available, we will have more sufficient evidence to make well-grounded suggestions for the further development and application of the tool in other (breast) cancer types.

## 5. Conclusions

The BC-ADA is acceptable and usable in supporting decision-making on personalized aftercare for breast cancer survivors. It holds promise for improving information provision, facilitating better conversations between patients and HCPs about care needs and co-creation of aftercare plans. Its current implementation will provide further insights into the BC-ADA’s cost-effectiveness and potential to promote future implementation in more hospitals.

## Figures and Tables

**Table 1:** Members of the steering group (N = 21).

Discipline	Number
Surgical oncologist	3
Nurse specialist	4
Nurse practitioner	1
Radiologist	2
Psychologist	1
General practitioner	2
Patient representative	2
Researcher	4
Web developer	2

**Table 2:** Potential benefits of the PtDA, according to patients (N = 18) and HCPs (N = 15).

According to Patients	N	Example Quote
Prepares for consultation	8	“*Before you see her [HCP], that you fill in the tool. For me, that would be easier than having to lay everything out on the table at that moment.*”
Provides specific information	7	“*That you can click directly on the topic and know where to look, because searching can take a long time.*”
Easy access to reliable information	3	“*You can Google a lot, but you find so much that isn’t true. So, if you have it all in one place where you know it’s reliable information, I would have appreciated that.*”
Information for family	2	“*That I can tell my children: ‘Guys, here you can read what this does to me or what I’m struggling with. That way, you might better understand how I feel’*”
**According to HCPs**		
Prioritizes patients’ main care needs	8	“*Self-reflection is naturally more beneficial than if I gave them a symptom checklist to fill out on the spot. So, I can definitely see some value in that preparatory step*”
Activates patients to engage in their own recovery	8	“*If the patient has considered for themselves, ‘This is something I would like to work on,’ you have a much higher chance of success than if we recommend something*”
Information consolidated into one tool	6	“*It’s especially important to have one place or website where patients can find all information, so that you can easily refer them to a single source*”
Information normalizes and reassures patients’ experiences	2	“*That patients read that these things are actually normal and not necessarily a problem that requires intervention. That alone reduces anxiety or stress, and consequently, reduces hospital visits*”
Information prevents future health problems	3	“*Education can have a very preventive effect on health problems, even in the future*”
Aftercare plan facilitates communication with the GP *	4	“*It’s certainly beneficial if, when they go to a GP, the GP is informed about what has occurred and what is ongoing*”

* General Practitioner.

## Data Availability

The datasets generated during and/or analysed during the current study are not publicly available due to participants’ privacy but are available from the corresponding author on reasonable request.

## Supplementary Materials

The following supporting information can be downloaded at: https://www.mdpi.com/article/10.3390/curroncol32100552/s1, Figure S1: Handout sheet; Figure S2: Online tool_information domains; Figure S3: Online tool_information practical tips; Figure S4: Online tool_questions about goals; Figure S5: Summary sheet; Table S1: Patient and HCP reported requirements in needs assessments, information provision and aftercare plans; Table S2: Positive and negative comments on usability and subsequent changes in the tool.

## Abbreviations

The following abbreviations are used in this manuscript:

BC-ADA	Breast Cancer Aftercare Decision Aid
HCP	Healthcare professional
NP	Nurse practitioner
NS	Nurse specialist
PtDA	Patient decision aid
SDM	Shared decision-making

## Appendix A. Interview Scheme for Healthcare Professionals

Part 1: Assessment of care needs - How satisfied are you with the way complaints, needs, and the patient’s situation are assessed? ο Could this be improved? If so, in what way? - To what extent do you have enough information from the patient to assess their aftercare needs? ο What information do you use for this assessment? ο What do you feel is missing when assessing the patient’s complaints and needs? ο What change do you expect from this addition (i.e., what is currently missing)? *Interviewer explains:* *There are also various tools to assess complaints and care needs, such as the Distress Thermometer, mini-PROMs, and the spider web of positive health.* - What is your experience with these tools, and what is your preference? - What considerations do you have? What advantages and disadvantages do you see? Part 2: Information provision *Interviewer explains:* *In aftercare, patients typically receive various information from healthcare providers, such as common complaints, available care and support in the hospital, assistance outside the hospital,* etc. - How do you evaluate the current information provided? How understandable, complete, and tailored is the information for your patient? - What topics do you consider important to inform the patient about? - What do you feel is missing in your current information delivery? Or: what would you like to see done differently? ο What change do you expect from this addition (i.e., what is currently missing)? - What information do you think is important for the patient to make an informed decision about how aftercare is structured? Part 3: Aftercare plan *Interviewer explains:* *The guidelines recommend using an aftercare plan: an overview containing aftercare information and agreements that the patient can easily keep at hand. At the beginning of aftercare, the patient can collaborate with the healthcare provider to create such a plan. For example, they might decide together what additional support is needed, what ongoing concerns should be addressed, and what follow-up consultations are required.* - What do you think of such an aftercare plan? - What should be included in the aftercare plan? What information do you consider important to provide to the patient in the aftercare plan? - What are your expectations for it? Do you foresee any advantages or disadvantages? Part 4: Barriers and facilitators for implementation of a supporting tool *Interviewer explains:* *To support the personalization of aftercare, we could develop a tool. This tool could be introduced to the patient by the healthcare provider, after which the patient would use it at home. In the tool, the patient could, for example, read information and answer questions about their care needs. The tool could then generate a summary of the patient’s responses. This summary could be used in the next aftercare conversation to collaboratively develop a personalized aftercare plan with the patient.* - What is your general impression of such a tool? - To what extent does the concept of this tool align with other similar initiatives (e.g., tools, programs) in your hospital? Or: how does this tool complement those? ο What are the potential advantages of this tool compared to these other similar initiatives? ο What are the potential disadvantages of this tool compared to these other similar initiatives? - How well do you think this implementation will meet the patients’ needs? ο What changes do you expect for the patient? Where do you see the greatest benefits for the patient? ο How do you expect patients to respond to the implementation? - What barriers do you anticipate patients will face when using the tool and making aftercare decisions? - How does the implementation of the tool fit with your current way of working? ο How do you expect the tool to integrate into the current workflow? ο What will it change in your current practice? Which aspects will the tool replace? - What changes or adjustments do you think are necessary within your organization to effectively use this tool and create personalized aftercare plans? ο Are these changes/adjustments feasible? - To what extent is the implementation of the tool and the aftercare plan feasible in terms of time investment and scheduling of consultations? - What aspects of your organization could hinder or facilitate the implementation? ο What can be done to prevent or reduce these hindrances? - Which team members are crucial to involve in this implementation? Or: Which team members need to support and be trained for successful implementation? ο To what extent are these team members positive about or willing to support the implementation? - How does this innovation align with the hospital’s mission/vision or national/international developments? - What motivates you to implement this tool successfully? - How confident are you that you will be able to implement the tool successfully? ο What gives you this sense of (un)certainty?
