# Supplementary material for: A Goal Without a Plan Is Just a Wish—Creating a Personalized Aftercare Plan for Breast Cancer Patients Supported by the Breast Cancer Aftercare Decision Aid
Source: Curr Oncol. 2025 Oct 1;32(10):552. doi: 10.3390/curroncol32100552 (PMC12564066; doi:10.3390/curroncol32100552)
Supplement: Supplementary file 1 [file curroncol-32-00552-s001.zip › Suppl. Tab. S1 S2.pdf]

Table S1. Patient and HCP reported requirements in needs assessments, information provision and aftercare plans

Requirement	P	HCP	Incorporated in the tool
**Assessment of care needs**			
Provides means to prioritize care needs	X	X	Module 5 includes questions about what domains patients wish to improve first.
Uses positive health approach	X	X	Module 2 and 5 include questions focusing on the patient's desired situation and goals, as well as how to achieve them.
Assesses needs on multiple domains	X	X	Module 3 provides information on multiple domains, and in Module 5, patients are asked which domain(s) they wish to improve.
Assesses need for support	X		Module 5 contains questions about the needed support from HCPs or social network, or wish to address issues independently.
Provides space for elaboration on care needs		X	In Module 5, patients are asked to elaborate on their goals, how they plan to achieve them, and where they require assistance.
Generates overview of care needs for patient and HCP		X	Patients' care needs are filled in in Module 5 and presented in the summary of Module 6, which can be shared with the HCP.
**Information provision**			
Informs about aftercare trajectory	X	X	The information sheet explains the aim of aftercare, and Module 1 contains information about the aftercare trajectory.
Informs about impact on multiple domains	X	X	Module 3 contains information on five domains of: physical recovery, daily life and work, emotions, bodily trust, and relationships. Sexuality is integrated into the domains of body image concerns and relationships due to its strong relevance to both.
Provides practical tips to self-manage complaints	X	X	In Module 3, tips are provided on how to prevent and treat complaints, and whom to approach for further help.
Informs about support options per domain	X	X	Each domain in Module 3 offers details on support options within and outside the hospital.
Provides links to other reliable information and websites	X	X	Module 3 includes 'read more' sections which provide links to additional resources, such as Dutch websites of the breast cancer patients organization and Kanker.nl, local support networks, websites about nutrition and lifestyle, and self-help apps.
Provides information tailored to received treatment	X	X	In Module 3, the description of common complaints specifies their potential causes, linking them to prior treatments.
Provides information also applicable to men	X		Gender-specific experiences with complaints are mentioned in Module 3. Module 4 also includes a man's story.
**Aftercare plan (i.e. written summary of agreements)**			
Includes contact details of HCPs		X	Not incorporated – HCPs are advised to provide contact details to patients in line with their workflow
Includes most important actions that patients can do themselves	X	X	Based on the summary sheet, HCPs and patients together decide what actions are necessary. Actions could involve self-management (e.g., daily walking) or referral to another HCP (e.g., a physiotherapist or psychologist).
Is concise		X	The aftercare plan only summarizes the joint agreements on goals, actions, and follow-up appointments.
Can be evaluated and adapted	X	X	Goals and actions in the aftercare plan can be reviewed and adjusted over time, based on the patient's changing needs.
**General requirements for the tool**			
Usable in various care pathways and points in time		X	Tool is usable at varying timepoints during the posttreatment phase, as its' content remains relevant throughout patients' recovery.
Link to hospitals' electronical patient records		X	Not incorporated – an automatic link between the tool and the EPD was not feasible.
Account for patients with lower health literacy		X	The tool's content is written at a B1 reading level and verified by the "Makkelijk Lezen [Easy Reading]" foundation.

Note: P = patient; HCP = healthcare professional. The online tool is comprised of six modules, which are defined in Figure 3 in the main manuscript.

Table S2. Positive and negative comments on usability and subsequent changes in the tool.

Component		Comments (+/-)	P	HCP	Change
**Printed information sheet**	+	Clear instructions about use of the tool	X	X	N/A
	+	Appealing visualization of different domains	X	X	N/A
	-	Information is limited for patients who receive sheet by mail instead of during consultation		X	Added an explanation that patients can use the tool to indicate what they wish to improve, which supports the creation of an aftercare plan in the next consultation.
**Online tool**	+	Easily accessible	X	X	N/A
	+	Text is easy to read	X	X	N/A
	+	Attractive layout with a professional appearance	X	X	N/A
	+	Comprehensive information	X	X	N/A
	+	The amount of information is manageable due to 'read more' sections	X	X	N/A
	-	At start, not immediately clear that the tool consists of both information and questions	X		Added a description of the tool's content and usage to the beginning of the tool
	-	There are few illustrations in the 'Living with' section		X	Incorporated icons from the information sheet into the "Living With" module
	-	Sexuality also plays a role in adjusting to and coping with a changed body, and not necessarily in context of relationships		X	Added a dedicated description of sexuality in domain of 'regaining trust in body'
	-	Unclear whether patients have to read all information	X	X	Added recommendation in introduction screen of the tool to read information in accordance with own time and need
	-	The ALERT! tool might evoke distress for some users who prefer not to see symptom-related information		X	Moved the link to the ALERT! tool to the bottom of the page to make it less prominent
	-	A link to the website 'Voeding en Kanker' [Nutrition and Cancer] is missing		X	Included a link and explanation about the Voeding en Kanker [Nutrition and Cancer] website
	-	The use of a leg icon for "Edema" is confusing, as this mostly occurs in the arm		X	Replaced the leg icon for "Edema" with an arm icon
	+	Personal stories are relatable	X	X	N/A
**Summary sheet**	+	Care needs are displayed in a clear and organized manner	X	X	N/A
	+	Useful during consultation	X	X	N/A
	-	Option for sharing the summary to with the HCP prior to the consultation should be considered		X	Added a button to the module 'summary', allowing patients to send the summary directly to the HCP's mailbox
**General**	-	Concern about time investment when patients feel obliged to read all information		X	Updated instructions about introduction of the tool, emphasizing that HCPs should explain the importance of completing the questions, while clarifying that reading all information is only optional.

Note. P = patient; HCP = healthcare professional; N/A = not applicable
