# Supplementary figures and images for: A Goal Without a Plan Is Just a Wish—Creating a Personalized Aftercare Plan for Breast Cancer Patients Supported by the Breast Cancer Aftercare Decision Aid
Source: Curr Oncol. 2025 Oct 1;32(10):552. doi: 10.3390/curroncol32100552 (PMC12564066; doi:10.3390/curroncol32100552)

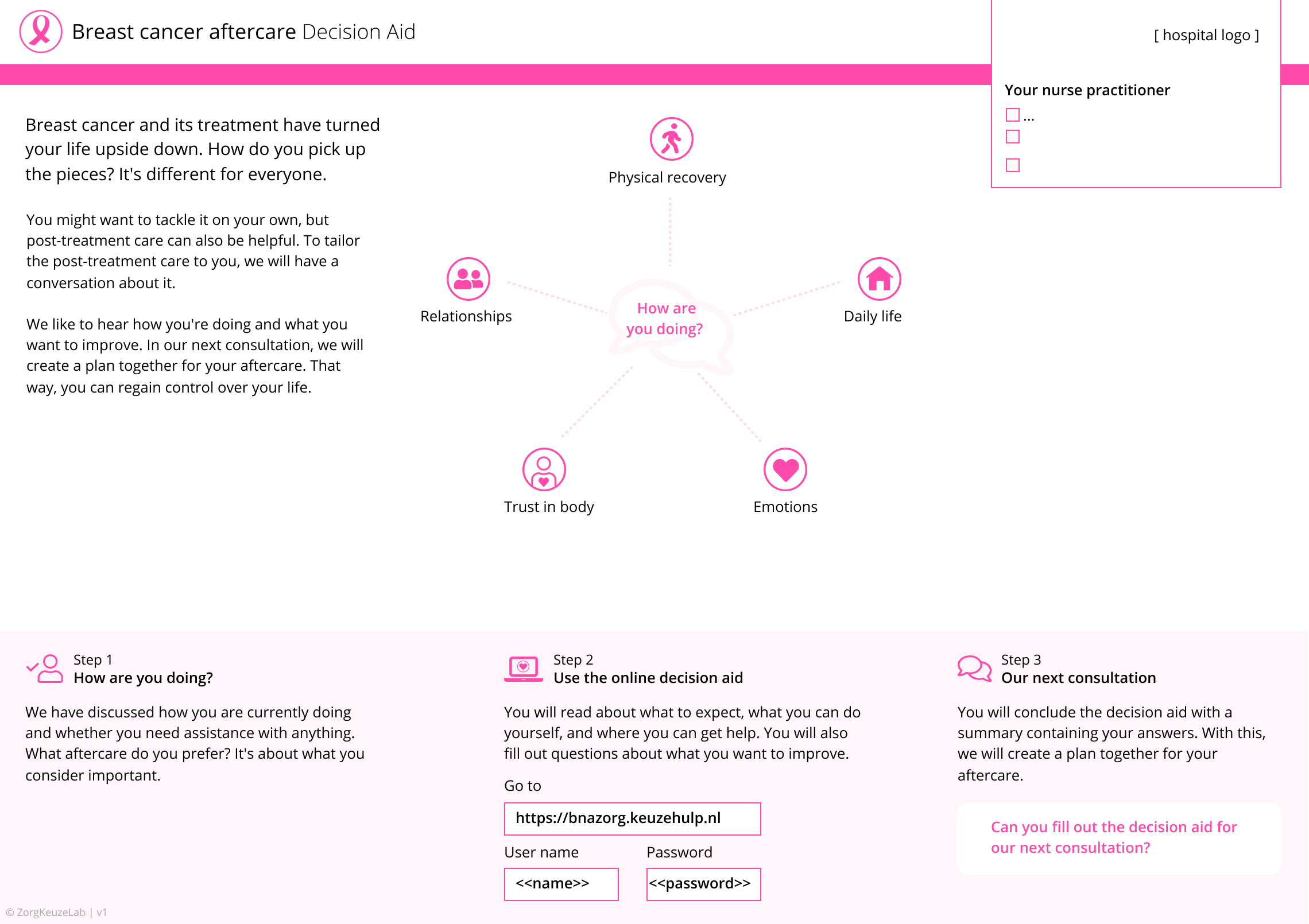

Supplement: Supplementary file 1 [file curroncol-32-00552-s001.zip › Suppl. Fig. S1_Handout sheet.png]

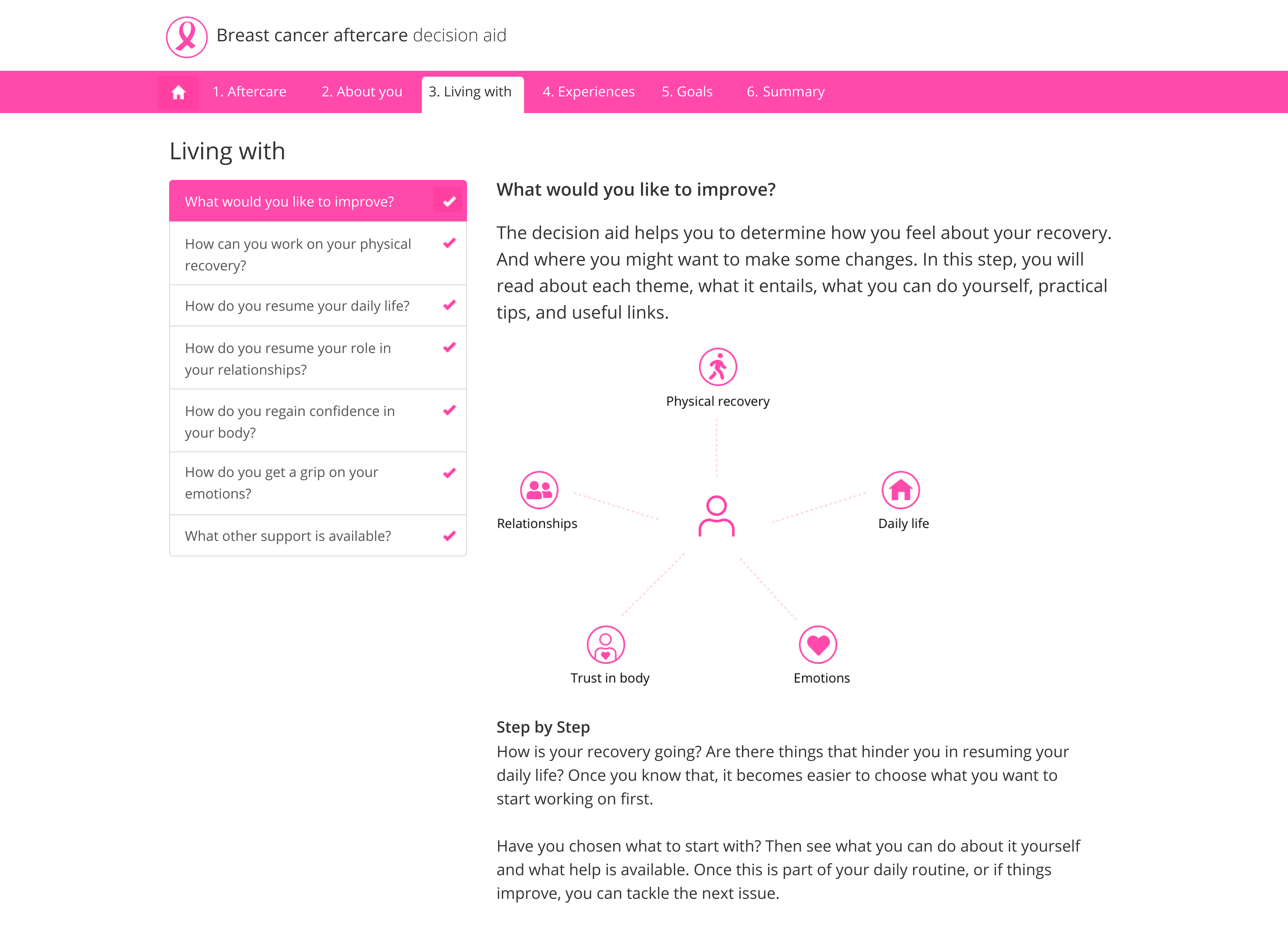

Supplement: Supplementary file 1 [file curroncol-32-00552-s001.zip › Suppl. Fig. S2_Online tool_information domains.png]

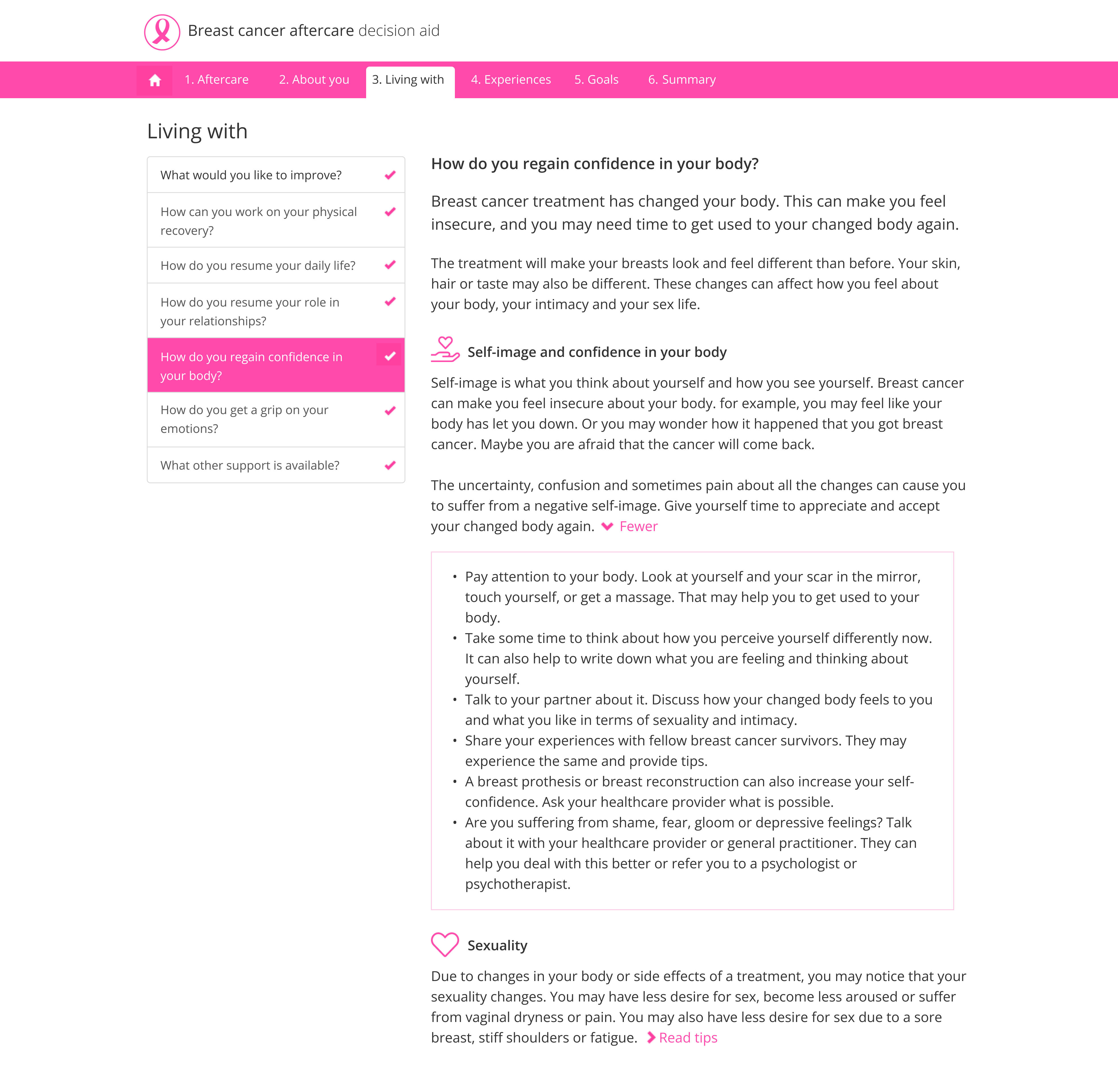

Supplement: Supplementary file 1 [file curroncol-32-00552-s001.zip › Suppl. Fig. S3_Online tool_information practical tips.png]

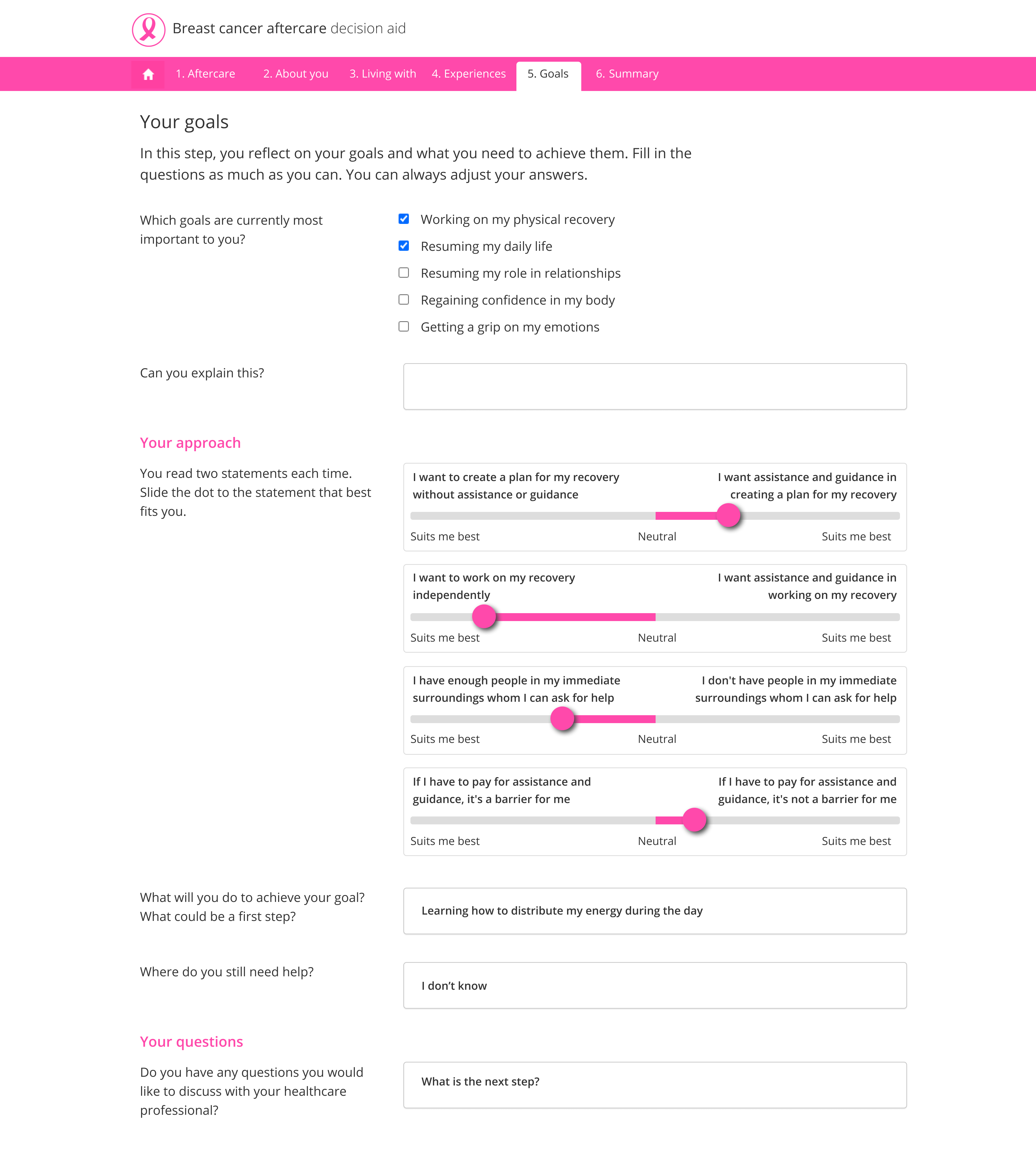

Supplement: Supplementary file 1 [file curroncol-32-00552-s001.zip › Suppl. Fig. S4_Online tool_questions about goals.png]

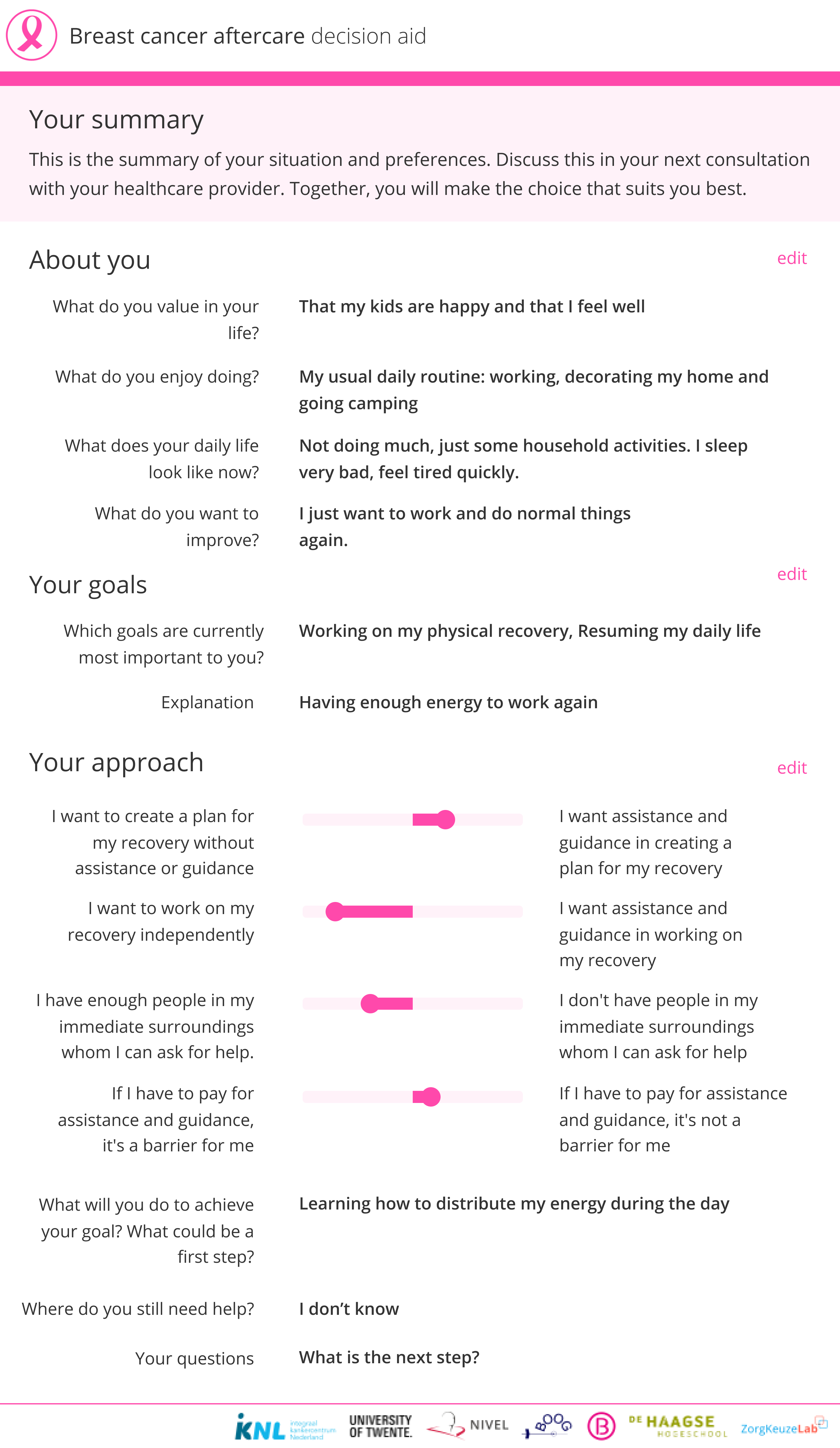

Supplement: Supplementary file 1 [file curroncol-32-00552-s001.zip › Suppl. Fig. S5_Summary sheet.png]
